# Cellular Response to Vitamin C-Enriched Chitosan/Agarose Film with Potential Application as Artificial Skin Substitute for Chronic Wound Treatment

**DOI:** 10.3390/cells9051185

**Published:** 2020-05-10

**Authors:** Vladyslav Vivcharenko, Michal Wojcik, Agata Przekora

**Affiliations:** Department of Biochemistry and Biotechnology, Medical University of Lublin, Chodzki 1 Street, 20-093 Lublin, Poland; vlad.vivcharenko@gmail.com (V.V.); michal.wojcik@umlub.pl (M.W.)

**Keywords:** biocompatibility, cell migration, proliferation, fibroblasts, keratinocytes, dermal skin grafts, epidermal skin grafts, drug release profile

## Abstract

The treatment of chronic wounds is still a meaningful challenge to physicians. The aim of this work was to produce vitamin C-enriched chitosan/agarose (CHN/A) film that could serve as potential artificial skin substitute for chronic wound treatment. The biomaterial was fabricated by a newly developed and simplified method via mixing acidic chitosan solution with alkaline agarose solution that allowed to obtain slightly acidic pH (5.97) of the resultant material, which is known to support skin regeneration. Vitamin C was immobilized within the matrix of the film by entrapment method during production process. Produced films (CHN/A and CHN/A + vit C) were subjected to comprehensive evaluation of cellular response with the use of human skin fibroblasts, epidermal keratinocytes, and macrophages. It was demonstrated that novel biomaterials support adhesion and growth of human skin fibroblasts and keratinocytes, have ability to slightly reduce transforming growth factor-beta 1 (TGF-β1) (known to be present at augmented levels in the epidermis of chronic wounds), and increase platelet-derived growth factor-BB (PDGF-BB) secretion by the cells. Nevertheless, addition of vitamin C to the biomaterial formulation does not significantly improve its biological properties due to burst vitamin release profile. Obtained results clearly demonstrated that produced CHN/A film has great potential to be used as cellular dermal, epidermal, or dermo-epidermal graft pre-seeded with human skin cells for chronic wound treatment.

## 1. Introduction

Wound healing is a highly regulated and complex process, that is crucial for maintaining the role of the skin as an external barrier. A dozen regulatory factors are involved in the healing process, leading to the complete restoration of basic skin functions. When this process cannot progress beyond the inflammatory phase, proliferation of vascular endothelial cells and fibroblasts is precluded, which leads to the wound chronicity [1]. A series of different complications, such as long-term hospitalization, amputation, or even death, can appear as a consequence of delayed wound healing. Nowadays non-healing wounds represent 2% in a general population, generating increased healthcare costs related to chronic wound treatment, exceeding 50 billion dollars annually [2]. Historically, treatment of the skin wounds has been one of the most essential and basal medical practices in human development. Currently, there is an extensive body of literature focused on the novel and technologically more advanced treatment methods of the chronic wounds [3]. Common strategies in the conventional treatment of the chronic wounds include skin grafting using autografts, allografts, or xenografts. However, when injuries cover a serious area of the body surface, collection of native skin tissue for autologous skin grafting can become a challenging task. In this case, application of allografts or xenografts may be a solution, however it carries high risk of graft rejection or disease transmission. Due to the limited availability of healthy donor tissue and high risk of immune rejection, surgical procedures involving natural skin grafts are more often replaced with artificial skin substitute transplantation. Therefore, tissue engineering is an upcoming alternative to the traditional skin tissue regeneration strategy [3,4,5]. There is no doubt that dynamic development of tissue engineering for the last 20 years has resulted in the significant progress in regenerative medicine and thereby has created perspectives for innovative reconstruction of damaged tissues, including entire organs [6]. Recently, a great variety of natural, synthetic, and bioengineered skin substitutes have been developed, which may be transplanted into the injured skin area, promoting wound healing, supplying oxygen, reducing pain, and providing protection against microorganisms as an external barrier [4]. 

Commercially available skin substitutes marketed for temporary or permanent use may be classified into three main types: epidermal, dermal, and dermo-epidermal grafts. Epidermal skin constructs are usually made of a keratinocyte sheet, which is prepared by cells isolation from the patient skin and their expansion under in vitro conditions. Modern epidermal skin grafts are produced by seeding the keratinocytes onto the surface of a thin membrane made of biomaterial to generate a cellular epidermal layer of the skin [7]. Acellular (without the cells) epidermal skin substitutes have a form of thin film (e.g., composed of polyurethane or silicone) that acts as an external barrier to cover the wound as well as to protect against dehydration and infections [8]. Despite the fact that preparation of cellular epidermal substitutes is often a long-term process, the grafts are fragile to handle, keratinocytes attachment is poor, and the production costs are high, they are still irreplaceable in the treatment of full-thickness skin defects and extensive burn wounds [7]. When it comes to dermal skin substitutes, they are frequently fabricated as permanent skin replacements made of polymer matrix without cells incorporated. The role of acellular dermal grafts is to facilitate infiltration of blood vessels and cells from the patient tissue, accelerating skin regeneration. The greatest advantages of such dermal substitutes are their easy storage, low immunogenicity, and low fabrication costs due to the lack of cells [7,9]. Some of the commercially available dermal skin substitutes are cellular and contain human fibroblasts, which release collagen, cytokines, and growth factors, accelerating re-epithelialization and the wound healing process [10]. Skin substitutes that are composed of two layers of the skin are known as dermo-epidermal grafts. They can be acellular or cellular with fibroblasts and keratinocytes incorporated. Cellular dermo-epidermal grafts are the most effective substitutes in the treatment of full-thickness skin defects and chronic ulcers since they provide cytokines and growth factors that are crucial for regulation of wound healing process and re-epithelialization [9,11]. 

The aim of this work was to produce chitosan/agarose (CHN/A) film enriched with vitamin C for potential application as artificial skin graft for chronic wound treatment. The biomaterial was fabricated using new simplified production method by neutralization of chitosan solution in acetic acid (CH_3_COOH) with agarose solution in sodium hydroxide (NaOH). Since neutralization of chitosan solution prepared in CH_3_COOH with NaOH—to pH higher than 6.5—is a common procedure to obtain chitosan gel, the concentrations of chitosan and agarose as well as CH_3_COOH and NaOH were optimized to receive slightly acidic pH (approx. 6.0) of the resultant mixture and to avoid uncontrolled precipitation (gelation and aggregation) of the chitosan component, which is known to be soluble at pH < 6.5. Obtained slightly acidic pH of the CHN/A film will not only allow for cell survival upon biomaterial seeding with the fibroblasts/keratinocytes in vitro which is the main concept of skin tissue engineering, but will also support skin regeneration upon potential biomaterial transplantation into the wound site. There are some papers in the available literature presenting chitosan/agarose wound dressings that were produced using completely different techniques compared to the method applied here. Most of the articles present foam-like biomaterials fabricated using a lyophilization process. For instance, Felfel et al. produced 3D chitosan/agarose scaffold for potential soft tissue repairing by freeze-drying technique [12]. Similarly, Rajesh et al. developed chitosan/agarose scaffolds for tissue engineering applications by freeze-drying followed by material stabilization in NaOH [13]. Hu et al. prepared chitosan/agarose film with potential biomedical applications by mixing chitosan solution in acetic acid with agarose solution in water, followed by its drying at 60 °C and subsequent freeze-drying [14]. Whereas, Miguel et al. developed chitosan/agarose hydrogel for skin regeneration by direct dissolution of agarose powder in acidic chitosan solution heated to 50 °C [15]. None of the above-mentioned production processes included neutralization of acidic chitosan solution with alkaline agarose solution to obtain the optimal for skin regeneration slightly acidic pH with no aggregation effect of chitosan component.

Vitamin C is a strong antioxidant and is known to be very important for the wound healing process as it promotes fibroblast proliferation, migration, and collagen deposition [2]. Therefore, vitamin C was immobilized within the polysaccharide matrix of the CHN/A biomaterial by entrapment method to provide antioxidant activity and to improve biological properties of the resultant biomaterial, what is especially important for the treatment of chronic wounds showing high levels of reactive oxygen species (ROS) and delayed healing [16,17]. Importantly, in the literature, most described methods for the production of chitosan/agarose biomaterials preclude entrapment of bioactive agents that are sensitive to extreme pH (acidic or alkaline). For instance, commonly applied neutralization of chitosan/agarose blend with NaOH after the production process precludes vitamin C addition because of its fast oxidation in alkaline environment [18]. It is worth highlighting that the method for the production of CHN/A film was described in the Polish patent application no. P.430458. 

In our other studies, it was demonstrated that novel CHN/A film has high exudate absorption capacity, is prone to enzymatic biodegradation in the presence of lysozyme and collagenases, and reveals high elastic deformation enabling its adjustment to the wound area. The primary aim of this research was to assess cellular response to fabricated vitamin C-enriched CHN/A film. Therefore, produced biomaterials (with and without vitamin C) were subjected to comprehensive evaluation of cytotoxicity, proliferation, growth, and migration of fibroblasts and keratinocytes, release of matrix metalloproteinases (MMPs) and growth factors (GFs) by fibroblasts, keratinocytes, and macrophages as well as production of type I collagen by fibroblasts. All these tests allowed to comprehensively determine biocompatibility and biomedical potential of vitamin C-enriched CHN/A film as artificial skin substitute for chronic wound treatment. 

## 2. Materials and Methods

### 2.1. Preparation of the Biomaterials

The 3% (*w/v*) chitosan solution (1.174 × 10^6^ Da molecular weight, kindly obtained from National Marine Fisheries Research Institute, Gdynia, Poland) in 0.5% (*v/v*) CH_3_COOH (Avantor Performance Materials, Gliwice, Poland) was mixed 1:1 with 4% (*w/v*) agarose solution (low EEO, Sigma-Aldrich Chemicals, Warsaw, Poland) in 0.1% (*w/v*) NaOH (Avantor Performance Materials, Gliwice, Poland) that was obtained by heating at 95 °C for 10 min on a magnetic stirrer. The pH of the chitosan/agarose mixture was assessed using pH meter equipped with ERH-11S electrode (Zabrze, Poland) intended for use with highly viscous solutions. Afterwards, 1/2 amount of the entire mixture was spread on the flat polystyrene surface of a Petri dish with 100 mm in diameter to form a thin layer (whole surface was covered by the blend) and left to air dry at room temperature. The resultant film was marked as CHN/A throughout the manuscript. The remaining half of the mixture was cooled to 40 °C and 3-*O*-ethyl-L-ascrobic acid (vitamin C) solution (Sigma-Aldrich Chemicals, Warsaw, Poland) prepared in phosphate buffered saline (PBS, Sigma-Aldrich Chemicals, Warsaw, Poland) was added to obtain final vitamin C concentration equal to 100 µg per 1 mL of the chitosan/agarose mixture (the highest nontoxic vitamin C concentration was applied that was selected during pilot cytotoxicity studies). The chitosan/agarose blend was mixed with the vitamin C on the magnetic stirrer to provide uniform distribution of ascorbic acid within biomaterial structure. It should be noted that 3-*O*-ethyl-l-ascrobic acid is a form of vitamin C revealing high stability at high temperatures, which does not need to be protected from light and oxygen. Then, the pH of the mixture was checked and obtained mass was spread on the polystyrene surface and left to dry as described above. Biomaterial containing vitamin C is referred to as CHN/A + vit C throughout the manuscript. Biomaterial with a thickness of approx. 2 mm in a wet state was produced by spreading 15 mL of the blend on the Petri dish with 100 mm in diameter, whereas film with the thickness of 0.15 mm was prepared by spreading 5 mL of the chitosan/agarose mixture. The thickness of the films was measured using electronic micrometer (measurement range: 0–25 mm, accuracy: 0.001 mm, Schut Geometrical Metrology, Groningen, The Netherlands).

### 2.2. Vitamin C Release Profile 

The release profile of vitamin C from the CHN/A + vit C sample (weighing 250 mg) was examined using a flow-through procedure in a closed-loop system. The experiment was carried out in the USP4 Sotax drug release apparatus (Donau Lab, Switzerland) using 50 mL of PBS as an elution medium, which circulated at a rate of 3 mL per min at 37 °C. At specified time intervals, 0.5 mL samples were collected for the evaluation of vitamin C concentration. In order to maintain the original volume of the elution medium, the system was refilled with the same capacity of the fresh PBS. Vitamin C concentrations were determined by measurement of absorbance values at a wavelength of 252 nm using a UV-spectrophotometer (Genesys 6 UV-Vis, Thermo Fisher Scientific, Waltham, MA, USA). A calibration curve was prepared for known concentrations of vitamin C solutions, which were prepared in PBS in the range of 3.75–60 μg/mL. The vitamin C release profile from the CHN/A + vit C biomaterial was presented as cumulative concentration at defined time intervals. 

### 2.3. Cell Culture Tests

In the present work, normal human skin fibroblasts (BJ), normal human epidermal keratinocytes transformed with HPV-16 (HEK001), and human acute monocytic leukemia cells (THP-1) obtained from American Type Culture Collection (ATCC-LGC Standards, Teddington, United Kingdom) were used. BJ cells were cultured in Eagle’s Minimum Essential Medium (EMEM, ATCC-LGC Standards, Teddington, United Kingdom) with a 10% fetal bovine serum content (FBS, Pan-Biotech GmbH, Aidenbach, Bavaria, Germany). HEK001 cells were cultured in Keratinocyte-Serum Free medium with 2 mM L-glutamine and 5 ng/mL human recombinant EGF content (Gibco, Life Technologies, Grand Island, NY, USA) to obtain complete culture medium. THP-1 cells were cultured using Roswell Park Memorial Institute medium (RPMI, ATCC-LGC Standards, Teddington, United Kingdom) with the addition of 10% FBS, 0.05 mM 2-mercaptoethanol, and 200 nM of phorbol myristate acetate (PMA) (Sigma-Aldrich Chemicals, Warsaw, Poland), which induced THP-1 differentiation to mature macrophages. All media were additionally supplemented with penicillin (100 U/mL) and streptomycin (100 µg/mL) obtained from Sigma-Aldrich Chemicals (Warsaw, Poland). Culture conditions for the all cell lines were the same: 37 °C, 95% of air humidity, and 5% CO_2_. Cells were subcultured when the confluence reached 90%–95%.

#### 2.3.1. Cytotoxicity Test

Direct contact cytotoxicity test was used to evaluate BJ fibroblasts viability on the surface of the films. Biomaterials were cut into small square samples (7 × 7 mm), placed in a 48-multiwell plate and presoaked in EMEM medium. Then, BJ cells were seeded on the top surface of the biomaterials in 500 µL of the culture medium at a concentration of 1 × 10^5^ cells per sample. After 48-h culture at 37 °C, the BJ cells were stained using Live/Dead Double Fluorescent Staining Kit (Sigma-Aldrich Chemicals, Warsaw, Poland) and analyzed using confocal laser scanning microscope (CLSM, Olympus Fluoview equipped with FV1000, Olympus, Corporation, Tokyo, Japan).

#### 2.3.2. Cell Growth on the Biomaterials

Cell growth and proliferation on the surface of the films were determined with use of human skin fibroblasts (BJ cells) and epidermal keratinocytes (HEK001). The cells were seeded directly on the presoaked samples of the biomaterials (which were placed in a 48-multiwell plate), in 500 µL of the culture medium at a concentration of 1 × 10^4^ cells per sample. Cells cultured on the surface of tissue culture-treated glass coverslip served as a control. The cells were cultured for 2, 4, and 6 days at 37 °C (half of the culture medium was replaced with fresh portion every 2–3 days), and then cell number on the surface of the film was determined using Cell Counting kit-8 (WST-8 test, Sigma-Aldrich Chemicals, Warsaw, Poland) as it was described earlier [19]. 

Due to the fact that the WST-8 is non-toxic to the cells, after a quantitative evaluation of cell number on the 6th day, the same cells were fixed and visualized by fluorescent staining of their cytoskeleton and nuclei. Briefly, the cells were fixed in 3.7% formaldehyde solution for 10 min, permeabilized with 0.2% TritonX-100 for 15 min, and blocked with 1% bovine serum albumin (all reagents were purchased from Sigma-Aldrich Chemicals, Warsaw, Poland). The cells were stained for 30 min at room temperature using 0.5 µg/mL DAPI solution (Sigma-Aldrich Chemicals, Warsaw, Poland) which gives blue fluorescence of nuclei and two units of AlexaFluor635-Phalloidin (Invitrogen, Carlsbad, California, USA) providing red fluorescence of F-actin filaments. 

To determine whether biomaterial may act as dermo-epidermal skin graft seeded with fibroblasts and keratinocytes, co-culture of BJ and HEK001 cells on the surface of the CHN/A film was performed for 6 days. The cells were fixed using the same protocol as described above and stained using DAPI, AlexaFluor635-Phalloidin, and anti-vimentin monoclonal antibody conjugated to AlexaFluor488 applied at a concentration of 1 µg/mL, giving green fluorescence of vimentin—a marker of mesenchymally-derived cells that is highly expressed in fibroblasts. Stained cells were observed using CLSM.

#### 2.3.3. Cell Migration

Cell migration was determined by wound healing assay (known also as scratch assay). Briefly, BJ and HEK001 cells were seeded into wells of 24-multiwell plates at a concentration of 1 × 10^5^ cells per well and cultured for 48 h to reach 95%–100% confluence. The cells were then vertically scratched using a sterile pipette tip and 500 µL of CHN/A and CHN/A + vit C extracts were added. The extracts were prepared according to ISO 10993-12 standard by immersion of the films in a complete culture medium (keeping the ratio 1 mL of the medium per each 15 mg of the sample) and their incubation for 24 h at 37 °C. Fresh culture medium was added to the control cells. After 24 h incubation, the cells were stained using May-Grunwald–Giemsa dye according to the procedure described previously [20]. Cell migration (wound/scratch closure) was determined based on the images obtained with inverted optical microscope (Olympus CKX53, Warsaw, Poland) using Photoshop software. The wound closure was calculated using the equation:

$$Wound\;Closure\;\%=\left[\frac{A_{0h}-A_{24h}}{A_{0h}}\right]\times 100$$

where *A*_0*h*_ is the area (pixels) of the wound calculated after scratching (*t* = 0 h) and *A*_24*h*_ is the area (pixels) of the unhealed wound (which is not covered by the cells) that remained 24 h after the scratching.

#### 2.3.4. MMP and GF Production

BJ fibroblasts, HEK001 keratinocytes, and THP-1-derived macrophages were seeded directly on the biomaterials as was described in Section 2.3.2. Cells cultured on the surface of tissue culture-treated glass coverslip served as a control. After 4-day culture, the cell culture supernatants were collected to determine concentrations of MMPs: (1) MMP-1, (2) MMP-2, (3) MMP-8 and GFs: (1) transforming growth factor-beta 1 (TGF-β1), (2) platelet-derived growth factor-BB (PDGF-BB). MMP and GF concentrations were assessed using ELISA kits specific to human MMP-1, MMP-2, MMP-8, TGF-β, and PDGF-BB (Biorbyt ELISA kit, Cambridge, United Kingdom), which were performed according to the manufacturer protocol.

#### 2.3.5. Type I Collagen Production

BJ cells were seeded directly on the biomaterials as was described in Section 2.3.2. The control samples were prepared by seeding the cells onto tissue culture-treated glass coverslip. Fibroblasts were cultured for 6 days with medium renovation every 3 days. The amount of type I collagen was assessed in cell lysates using ELISA kit specific to human type I collagen (Col I, EIAab ELISA kit, Wuhan, China). The cell lysates were prepared by application of freeze–thaw cycles and ultrasonication according to the procedure described earlier [21]. The level of produced collagen was normalized for each sample per 1 mg of cellular proteins determined by BCA Protein Assay Kit (Thermo Fisher Scientific, Waltham, MA, USA).

Type I collagen production was also evaluated qualitatively by immunofluorescence technique. Fibroblasts were fixed using the same method as described in Section 2.3.2. The samples were incubated overnight at 6 °C with primary human specific anti-type I collagen (Col1a1/Col1a2) antibodies (Abnova, Taipei, Taiwan) prepared at a concentration of 10 µg/mL. Then, the materials were washed with PBS and secondary antibodies conjugated to AlexaFluor647 (Abcam, Cambridge, United Kingdom) prepared at a concentration of 2 µg/mL were added. After 1 h incubation at room temperature, the samples were stained with 0.5 µg/mL DAPI and observed under CLSM.

### 2.4. Statistical Analysis 

Presented results were obtained from at least three independent experiments, and were shown as mean values ± SD. Statistical analysis of the results was performed using one-way ANOVA followed by Tukey’s test with a significance considered at *p* < 0.05 (GraphPad Prism 8.0.0 Software, GraphPad Software Inc., La Jolla, CA, USA).

## 3. Results and Discussion

### 3.1. Biomaterial Fabrication

**Figure 1:**
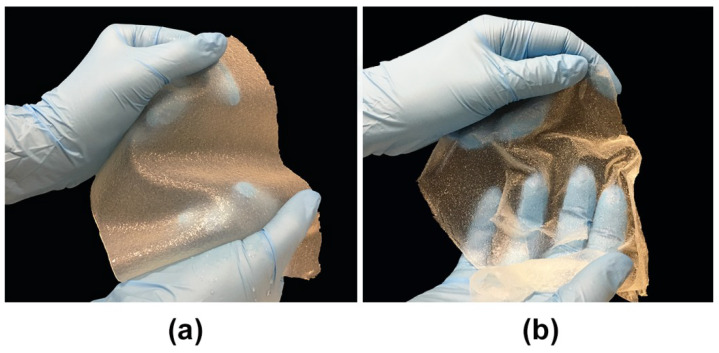
Images presenting fabricated chitosan/agarose (CHN/A) films: (**a**) CHN/A film in a wet state with a thickness of 2.12 mm (potential dermal skin graft); (**b**) CHN/A film in a wet state with a thickness of 0.15 mm (potential epidermal skin graft).

The epidermal layer of the skin that acts as a main skin barrier is known to be 0.07–0.12 mm thick, whereas dermal layer has usually a thickness of about 1–4 mm [22]. Applied within this research, a new method for the production of skin substitute allowed to obtain chitosan/agarose films with different thicknesses depending on the intended use, that revealed homogenous but rough surfaces (Figure 1). Importantly, the pH of the CHN/A mixture was slightly acidic and equal to 5.98, whereas CHN/A + vit C showed pH equal to 5.97. Therefore, addition of vitamin C at concentration of 100 µg per 1 mL of chitosan/agarose blend did not significantly affect pH of the resultant film. It is known that slightly acidic pH of the skin plays a significant role in the wound healing as it helps to control antimicrobial activity, angiogenesis, oxygen release, and protease activity [23]. Obtained slightly acidic pH values of the biomaterials, imitating natural skin pH, are beneficial for skin regeneration by promoting fibroblast proliferation and inhibiting MMP activity. Whereas, alkaline environment is harmful for the process of skin healing because MMPs show the highest activity at pH 8.0. Thus, chronic wounds due to the chronic inflammation have elevated pH and excessive MMP activity, leading to ECM degradation [24]. Moreover, chronic wounds are characterized by significantly decreased levels of tissue inhibitors of metalloproteinases (TIMPs) that regulate activity of MMPs. Thus, many therapeutic strategies include topical administration of protease inhibitors to the wound area to reverse its chronicity [25]. Nevertheless, it was demonstrated that by lowering the pH at the site of a chronic wound it is possible not only to inhibit MMP activity, but also to create adverse conditions for the infection development, and stimulate fibroblast divisions [25,26]. In this study the pH value of the biomaterials was adjusted to maintain high survival of the skin cells after their seeding onto the films. Thus, to avoid cytotoxicity of strongly acidic pH against skin cells, the pH of the films was kept close to 6.0. Maintenance of high viability of the cells on the surface of the produced films is crucial since the biomaterials were designed for potential application as artificial skin grafts pre-seeded with patient skin cells. It is also worth noting that pH value of newly formed epithelium at the wound edges is around 5.90 which is close to the pH value of developed films [24].

### 3.2. Vitamin C Release Profile 

Ascorbic acid (vitamin C) was confirmed to play a pivotal role in the chronic wound regeneration by its high antioxidant, anti-inflammatory, and skin-healing promotive properties. It is also engaged in the stimulation of fibroblast proliferation, collagen synthesis, and tissue remodeling. Thus, scorbutic patients experience delayed skin healing due to reduced rates of collagen synthesis and its maturation [2]. Since vitamin C deficiency leads to healing disorders, enrichment of the wound site with ascorbic acid may speed up the regeneration process, especially in the case of chronic non-healing wounds that are characterized by excessive ROS production and prolonged inflammation [16,17]. 

**Figure 2:**
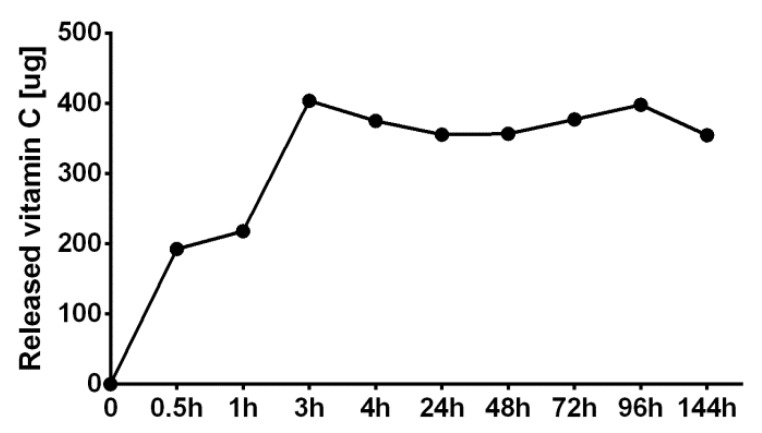
Cumulative vitamin C release profile from the CHN/A + vit C film.

Within this study, a variant of CHN/A film enriched with 3-o-ethyl-L-ascrobic acid was produced to provide accelerated healing process of chronic wounds. The release profile of the vitamin C from the CHN/A + vit C sample was tested in the closed-loop system using PBS as the elution medium. The film showed a high initial burst release of the vitamin C since already after 3 h the plateau effect was observed and further vitamin C release was not detectable (Figure 2). The best therapeutic effects are observed when the drug is released from the biomaterial at a constant rate for a prolonged period of time [27]. Therefore, controlled sustained vitamin C release from CHN/A + vit C film would ensure the most effective treatment of the chronic wounds. In this study, vitamin C was immobilized within polysaccharide chitosan/agarose matrix by simple physical entrapment method. The main limitation of this method is susceptibility to the leakage of the entrapped molecules [28]. 

### 3.3. Cytotoxicity Test 

**Figure 3:**
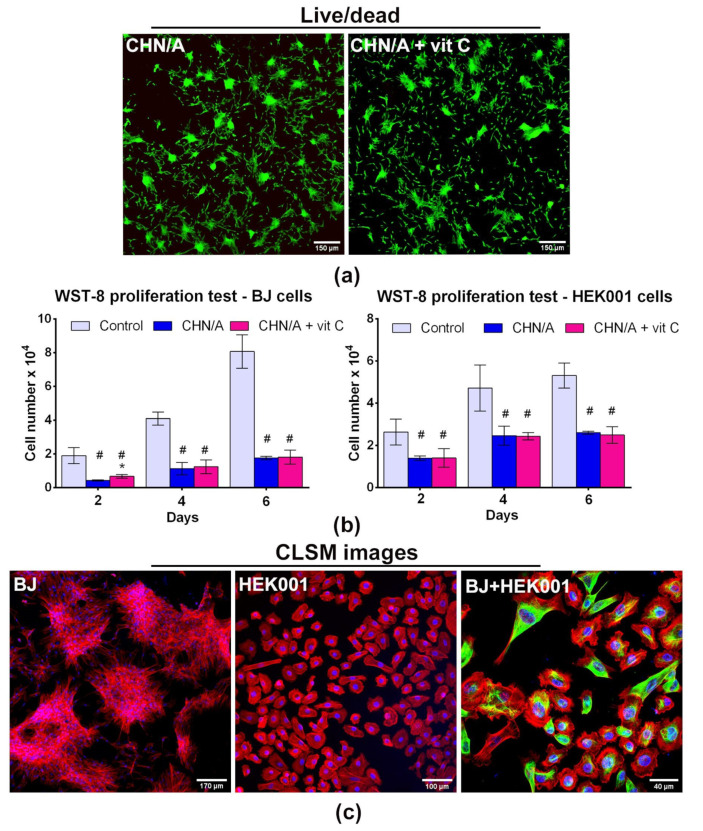
Biocompatibility of the films assessed with the use of human skin fibroblasts (BJ cell line) and human epidermal keratinocytes (HEK001 cell line): (**a**) cytotoxicity evaluation by live/dead staining of BJ cells cultured on the produced films for 48 h (viable cells emit green fluorescence, dead cells give red fluorescence); (**b**) BJ and HEK001 proliferation on the surface of the films assessed by colorimetric WST-8 test (control—cells cultured on the glass coverslip, * statistically significant results compared to CHN/A sample, **#** statistically significant results compared to the control cells; *n* = 5 (five independent samples were tested), *p* value < 0.05, one-way ANOVA followed by Tukey’s test); (**c**) BJ and HEK001 growth on the surface of CHN/A film visualized upon 6-day culture by fluorescent staining of nuclei (blue fluorescence) and actin filaments (red fluorescence); co-culture (BJ + HEK001) was additionally stained by immunofluorescence using anti-vimentin antibodies (green fluorescence) to distinguish fibroblasts (vimentin-positive) from keratinocytes (vimentin-negative or slightly positive).

To estimate cellular response to the obtained hydrogels, the CHN/A and CHN/A + vit C films were subjected to in vitro cell culture experiments with the use of human skin fibroblasts (BJ), human epidermal keratinocytes (HEK001), and human THP-1 cell line-derived macrophages. Biocompatibility of the skin substitute is a significant issue since biomaterials used as artificial skin grafts should be non-immunogenic, non-toxic, and supportive to cell proliferation [4]. Cytotoxicity of the produced biomaterials was determined by direct contact method using live/dead fluorescent staining of the BJ fibroblasts grown on the films. As shown in Figure 3a, live/dead staining of the cells cultured on the surface of the CHN/A and CHN/A + vit C films proved non-toxicity of the produced biomaterials. Obtained CLSM images showed numerous flattened, well attached, and viable (green fluorescence) fibroblasts on the surface of obtained films, indicating their high biocompatibility. Importantly, no dead cells (red fluorescence) were detected on the biomaterials.

### 3.4. Cell Proliferation and Growth 

Good cell adhesion and proliferation on the surface of the biomaterials is a crucial feature, enabling their use as cellular artificial skin grafts in regenerative medicine. Moreover, accelerated cell proliferation allows for speeding up the injury regeneration [29]. Since ascorbic acid is known to promote fibroblast proliferation [2], vitamin C-enriched biomaterial was fabricated to provide an improved healing process through enhancement of cell proliferation and growth. WST-8 proliferation test revealed an increase in BJ cell number with time on both films (CHN/A and CHN/A + vit C), indicating that developed biomaterials supported fibroblast proliferation (Figure 3b). Surprisingly, apart from the 2nd day of the experiment there were no statistically significant differences in BJ cell number on the CHN/A and CHN/A + vit C film. 

In the case of HEK001 keratinocytes, increase in cell number was observed only until the 2nd day of the experiment. After this time, the number of cells remained unchanged, which indicated that the cells were still viable and attached to the surface of the films but stopped their divisions. There were also no differences between the keratinocyte number on CHN/A and CHN/A + vit C film. According to the available literature it is known that keratinocytes require a relatively long time of culture to obtain a sufficient number of cells for transplantation or generation of bioengineered epidermal graft. This phenomenon is explained by the lack of crosstalk between fibroblasts and keratinocytes under in vitro conditions, which plays a key role in the promotion of keratinocyte proliferation in vivo [30]. Control cells grown on the tissue culture-treated glass coverslip showed significantly faster proliferation compared to the cells cultured on the films. It is a commonly observed phenomenon in the field of engineering of biomaterials since cells grown on the surface of the materials require more time for the Lag phase (adaptation to the growth substrate) what results in the delayed logarithmic (Log) growth phase. Whereas, cells grown on the optimal growth substrate (like tissue culture-treated polystyrene or glass) start the Log phase earlier and give better results in proliferation assays compared to the cells cultured on the biomaterial surface [31,32]. Nevertheless, performed CLSM imaging demonstrated good condition of the cells on the surface of the CHN/A film, proving that the developed biomaterials support skin cells growth (Figure 3c). From the obtained results, it may be concluded that the addition of the vitamin C to the developed film did not have a positive impact on cell proliferation, which was most likely related to burst vitamin C release from the CHN/A + vit C sample demonstrated in drug release test (Figure 2). As half of the culture medium was replaced with fresh portion every 2–3 days of the experiment (and thus ascorbic acid concentration was diluted), vitamin C concentration that is sufficient to promote fibroblast proliferation was reached only on the 2nd day.

Since addition of vitamin C to the biomaterial did not improve cell proliferation, fibroblast and keratinocyte growth was visualized only on the surface of CHN/A film (without vitamin C). Six days after cell seeding, fluorescent staining was performed to assess morphology and growth of fibroblasts and keratinocytes on the surface of the film. In the case of co-culture visualization, fibroblasts were double stained and exhibited actin-positive (red) and vimentin-positive (green) fluorescence of cytoskeleton, whereas keratinocytes showed only red fluorescence of actin filaments. However, it should be noted that single HEK001 cells were slightly vimentin-positive and emitted weak signal of green fluorescence, which was a result of their adaptation to the in vitro culture conditions [33].

Obtained CLSM images showed that cells grown on the surface of the biomaterial were well attached, had their typical morphology, and extensive cytoskeleton structure (Figure 3c). Thus, it was demonstrated that CHN/A film was favorable for growth of both cell types, including co-culture of fibroblasts and keratinocytes, which is very important taking into account that crosstalk and direct communication between fibroblasts and keratinocytes are crucial for the regulation of the skin regeneration process and wound re-epithelialization [30]. Performed experiment also proved that produced CHN/A biomaterial may be potentially used in regenerative medicine as dermal graft seeded with fibroblasts, epidermal substitute seeded with keratinocytes, or dermo-epidermal construct seeded with both types of skin cells [7].

### 3.5. Cell Migration

Migration of fibroblasts and keratinocytes from the wound edge to the injured area is pivotal for the normal healing process. The key role of the keratinocytes is to migrate to the wound site to close the defect in the epidermal layer of the skin. Therefore, chronic non-healing wounds often reveal dysfunction in keratinocyte migration [34]. The role of dermal fibroblasts in the skin healing process is to provide wound contraction, extracellular matrix (ECM) deposition, and tissue remodeling [35]. In this study, scratch wound healing assay was carried out to evaluate the impact of developed skin substitutes on the migration of BJ fibroblasts and HEK001 keratinocytes. The migration ability of BJ and HEK001 cells was presented as the percentage of the wound (scratch) area that after 24 h was covered by the cells.

**Figure 4:**
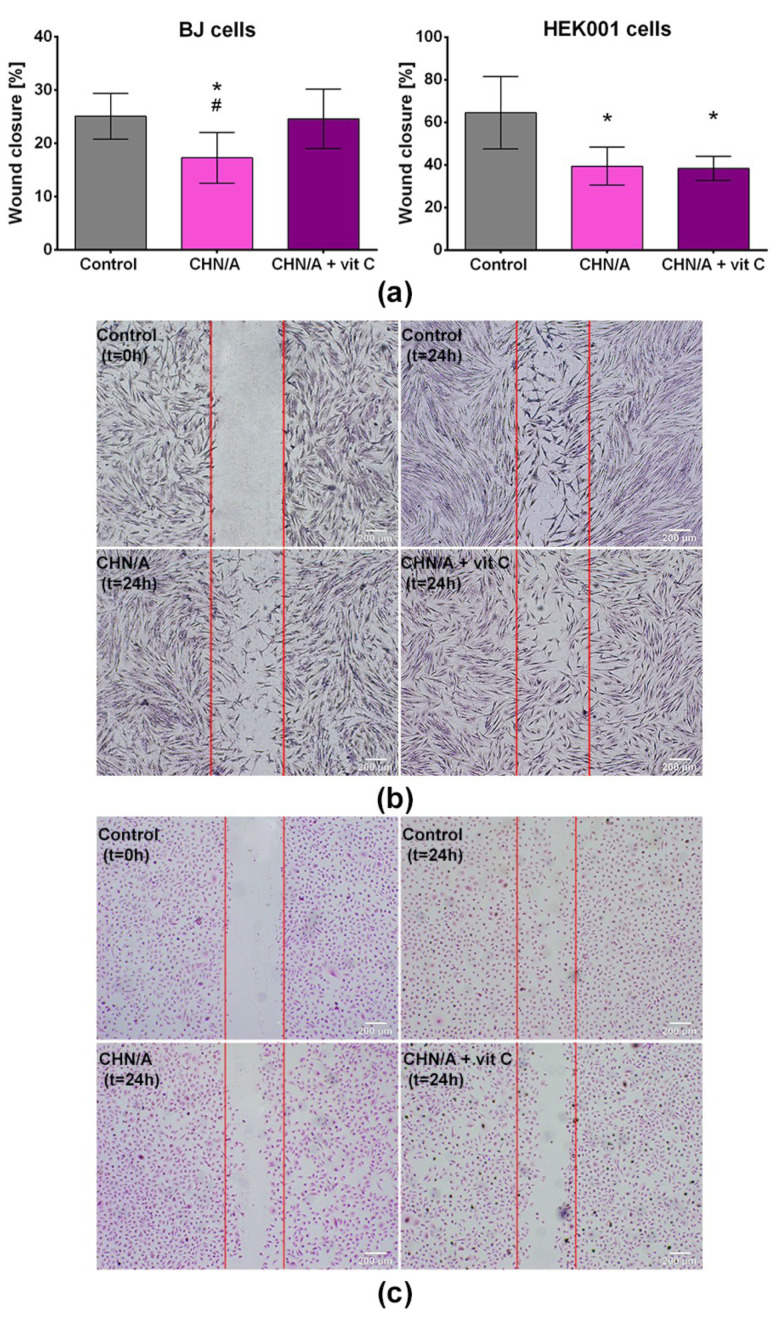
Cell migration determined by wound healing assay: (**a**) quantitative evaluation of wound closure (%) expressed as the percentage of the wound area covered by the cells 24 h after the scratching (* statistically significant results compared to control cells; # statistically significant results compared to CHN/A + vit C sample, *n* = 4 (four independent samples were tested), *p* value < 0.05, one-way ANOVA followed by Tukey’s test); (**b**) optical microscope images presenting scratch area covered by migrating BJ fibroblasts; (**c**) optical microscope images presenting scratch area covered by migrating HEK001 keratinocytes (control—cells cultured in the presence of culture medium, t_0_—wound area just after scratching, t_24h_—wound area 24 h after scratching).

Obtained results revealed that extract of CHN/A film slightly reduced migration of BJ cells (wound closure = 17%) compared to the control cells, however this negative effect was overcome by addition of vitamin C to the biomaterial (Figure 4a,b). The degree of wound closure determined for control cells and cells exposed to CHN/A + vit C extract was comparable and exceeded 24%. In the case of HEK001 cells, addition of vitamin C to the CHN/A film did not improve keratinocyte motility and the migration area reached approx. 38% for both tested biomaterials, which was significantly lower compared to the wound closure obtained with control cells (64%) (Figure 4a,c). By analyzing obtained results, it can be concluded that the addition of vitamin C to the CHN/A film had a positive effect on fibroblast (but not keratinocyte) migration by overcoming suppressive activity of CHN/A biomaterial. It is worth noting that CHN/A and CHN/A + vit C extracts generally allowed for cell migration into the scratched area, however this process was slowed down compared to the control cells. Therefore, although developed films were non-toxic and supported cell proliferation and growth (Figure 3), they failed in the aspect of stimulation of cell migration. Nevertheless, biomaterials did not completely inhibit motility of the cells, but slowed down this process, so this property does not exclude them from biomedical applications as skin substitutes.

### 3.6. MMP and GF Production

Matrix metalloproteinases (MMPs), which belong to the group of endopeptidases, are able to degrade main components of ECM [36]. MMPs may be secreted by numerous cells, including fibroblasts, keratinocytes, macrophages, and neutrophils [37]. MMPs and their inhibitors which are present in both chronic and acute wounds play a key role in the controlling ECM deposition and degradation that is fundamental for wound re-epithelialization. Thus, timed MMP activation at the wound site is essential for skin injury healing. Nevertheless, excessive protease activity may result in the formation of chronic non-healing wounds [37]. It was proven that fluids collected from the chronic wounds have significantly increased levels of elastase and MMPs, including MMP-1, MMP-2, MMP-8, MMP-9, and MMP-13 [38]. Taking into account that chronic wounds are characterized by elevated concentrations of MMPs, within this study ELISA tests were performed to determine whether developed films affect the synthesis of MMP-1, MMP-2, and MMP-8 by fibroblasts, keratinocytes, and macrophages. Gelatinase (MMP-2), type I and type II collagenases (MMP-1 and MMP-8, respectively) are enzymes largely engaged in the wound healing process. During normal wound regeneration, appropriate levels of MMP-1, MMP-2, and MMP-8 are crucial for skin healing, remodeling, and re-epithelialization. Nevertheless, it was shown that prolonged and augmented concentrations of active MMP-1, MMP-2, and MMP-8 cause a delay in epithelialization and are typical of chronic wounds [36]. Thus, downregulation of these enzymes seems to be a good strategy in the treatment of chronic non-healing wounds [37].

**Figure 5:**
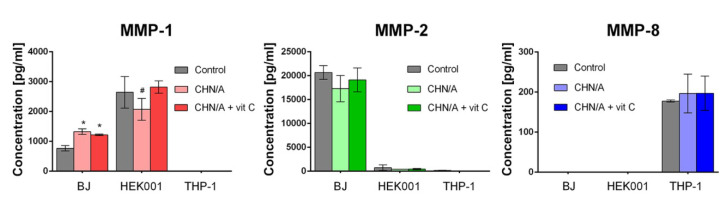
The level of metalloproteinases (MMPs) produced by human fibroblasts, keratinocytes, and macrophages cultured for 4 days on the surface of the films assessed by ELISAs (control—cells cultured on the glass coverslip, BJ—human skin fibroblasts, HEK001—human epidermal keratinocytes, THP-1—human macrophages differentiated from THP-1 cell line, * statistically significant results compared to the control cells; **#** statistically significant results compared to the CHN/A + vit C sample; *n* = 3 (three independent samples were tested), *p* value < 0.05, one-way ANOVA followed by Tukey’s test).

In this study, MMP secretion by the cells grown on the developed films was compared using three types of cells: fibroblasts (BJ cell line), keratinocytes (HEK001 cell line), and macrophages derived from THP-1 cell line. As shown in Figure 5, MMP-1, MMP-2, and MMP-8 production significantly varied depending on the cell type. The highest production of MMP-1 was detected for keratinocytes, whereas fibroblasts produced the highest amounts of MMP-2 among all cells used in the experiment. Macrophages were the only cells that had MMP-8 level detectable by ELISA. Synthesis of MMP-1 by macrophages was below detection range of ELISA used, whereas MMP-2 secretion by keratinocytes and macrophages was negligible. BJ fibroblasts cultured on the developed films revealed significantly increased production of MMP-1 compared to the control cells grown on the glass coverslip. Nevertheless, HEK001 keratinocytes grown on the CHN/A showed significantly decreased MMP-1 secretion compared to the CHN/A + vit C. Importantly, fibroblasts and keratinocytes cultured on the developed biomaterials showed slightly reduced MMP-2 secretion compared to the control cells, however the results were not statistically significant. MMP-8 release by macrophages cultured on the films was comparable to the level secreted by the control cells. Since the level of MMPs was not significantly elevated in contact with produced biomaterials (in some cases it was even slightly reduced), it may be inferred that produced films may be safely used for chronic wound treatment.

**Figure 6:**
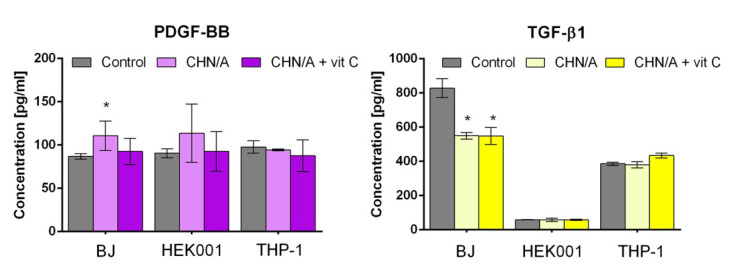
The level of growth factors (GFs) produced by human fibroblasts, keratinocytes, and macrophages cultured for 4 days on the surface of the films assessed by ELISAs (control—cells cultured on the glass coverslip, BJ—human skin fibroblasts, HEK001—human epidermal keratinocytes, THP-1—human macrophages differentiated from THP-1 cell line, * statistically significant results compared to the control cells; *n* = 3 (three independent samples were tested), *p* value < 0.05, one-way ANOVA followed by Tukey’s test).

Due to the fact that numerous GFs, including TGF-β, PDGF, keratinocyte growth factor (KGF), and many others, are engaged in the controlling the wound repair process [38], it was justified to also evaluate TGF-β1 and PDGF-BB synthesis by the cells seeded on the produced films. According to the ELISA results, PDGF-BB was secreted by all cell types (fibroblasts, keratinocytes, macrophages) at the similar level (87–114 pg/mL) regardless of the growth substrate—developed films or glass coverslip (Figure 6). However, CHN/A film showed tendency to slightly increase production of PDGF-BB by fibroblasts and keratinocytes, but obtained results were statistically significant only in the case of BJ cells. Since PDGF-BB is known to promote wound healing by stimulation of fibroblast proliferation and ECM deposition, some strategies for chronic wound treatment involve application of PDGF-BB delivery systems [39]. Therefore, it may be implied that observed augmented secretion of PDGF-BB by the cells grown on the CHN/A film is the desired phenomenon because it may provide accelerated healing of chronic wounds. 

In the case of TGF-β1, the highest production was observed for BJ cells and the lowest for HEK001 keratinocytes. Importantly, conducted ELISA test revealed that BJ fibroblasts cultured on the surface of the developed films secreted significantly lower amounts of TGF-β compared to the control cells. No statistically significant differences in the TGF-β1 production by keratinocytes and macrophages seeded on the films and glass coverslip (control) were observed. Among the variety of cytokines and GFs known to regulate the wound healing process, TGF-β exhibits the widest spectrum of different effects, including migratory and pro-proliferative roles by stimulation of endothelial and epithelial cell growth as well as fibroblast migration [40,41]. The conception of reduced TGF-β levels in human chronic wounds is most commonly described in the literature. Nevertheless, some reports identified augmented TGF-β levels in the epidermis of chronic wounds [41,42,43]. Therefore, it may be inferred that the artificial skin substitutes developed here with the ability to reduce the level of TGF-β may be effective in the treatment of chronic wounds.

### 3.7. Type I Collagen Production

**Figure 7:**
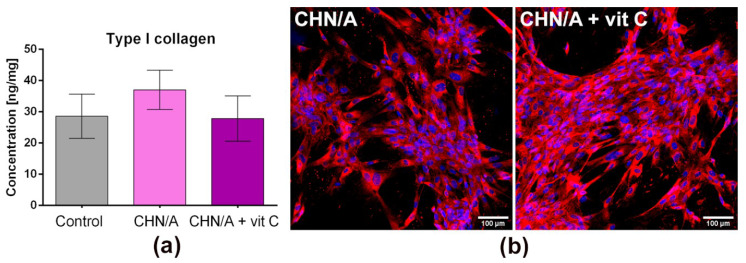
Results obtained with ELISA and immunofluorescence to detect the production of type I collagen by BJ fibroblasts grown on the surface of the films: (**a**) the level of type I collagen in the lysates of fibroblasts cultured for 6 days on the surface of the films assessed by ELISA; collagen synthesis was normalized per 1 mg of cellular proteins (control—cells cultured on the glass coverslip; *n* = 3 (three independent samples were tested)); (**b**) type I collagen deposited in the 6-day fibroblast culture performed on the surface of the films visualized by immunofluorescent staining (nuclei—blue fluorescence, type I collagen—red fluorescence).

Human fibroblasts secrete and arrange ECM, which supplies structural support for their migration, adhesion, and tissue organization [44]. ECM is primarily made of collagen, which provides elasticity and mechanical strength of the skin [4]. Deposition of the collagen fibers (type III and type I) by fibroblasts is one of the main steps during skin injury repair. However, excessive collagen production leads to the formation of unaesthetic scars [45]. Within this study, type I collagen deposition by fibroblasts cultured on the films was assessed by ELISA and immunofluorescent staining. Fibroblasts grown on the CHN/A biomaterial synthesized slightly greater amounts of collagen (approx. 37 ng/mg) compared to the cells cultured on glass coverslip (control sample) and CHN/A + vit C sample (approx. 27 ng/mg), nevertheless observed differences were not statistically significant (Figure 7a). Therefore, it may be implied that both produced hydrogels did not hinder collagen production by BJ cells, and provided its normal deposition as may be seen on CLSM images (Figure 7b). Interestingly, although ascorbic acid is a specific inducer of collagen production by fibroblasts [46], addition of vitamin C to the CHN/A film did not promote ECM deposition by BJ cells. This surprising result may be explained by burst vitamin C release profile from the CHN/A + vit C biomaterial and insufficient vitamin concentration (occurring after medium renewal during the experiment) to promote collagen synthesis.

## 4. Conclusions

Within this study it was demonstrated that CHN/A and CHN/A + vit C films fabricated by a new simplified method via mixing acidic chitosan solution with alkaline agarose solution exhibit slightly acidic pH (approx. 6.0), which not only enables maintenance of high viability of skin cells upon their seeding onto the biomaterials under in vitro conditions, but also is known to promote skin regeneration. It was demonstrated that novel biomaterials are non-toxic and support growth and proliferation of human skin fibroblasts and keratinocytes. Importantly, CHN/A film has ability to slightly increase PDGF-BB production and reduce TGF-β1 secretion (known to be present at augmented levels in the epidermis of chronic wounds), indicating its great potential to be used as artificial skin substitute pre-seeded with human skin cells for chronic wound treatment. Nevertheless, entrapment of vitamin C within polysaccharide structure of the film results in the burst release of the ascorbic acid and thus it does not significantly improve biological properties of the resultant biomaterial. Based on obtained results it may be concluded that produced CHN/A film has great potential to be used as cellular dermal, epidermal, or dermo-epidermal graft pre-seeded with human skin cells for chronic wound treatment. In order to more accurately estimate the biomedical potential of the produced hydrogels, the next scientific research step should involve in vivo experiments.
